# Teeth Receding in Vulcanization

**Published:** 1905-09

**Authors:** Geo. B. Snow

**Affiliations:** Buffalo, N. Y.


### TEETH RECEDING IN VULCANIZATION.

BUFFALO, N. Y., August 3, 1905.

TO THE EDITOR:

SIR,—On page 563 of the INTERNATIONAL DENTAL JOURNAL for August, there is a paragraph headed "Teeth receding in Vulcanization." As the advice given does not fit the case, may I venture to add something to it?

The phenomenon alluded to is a consequence of the shrinkage of rubber in vulcanizing. It often occurs when it is not suspected, and is the reason why, if it becomes necessary to remove a gum section from a vulcanite plate which has been worn, there will be found under it an exceedingly malodorous and nasty magma of particles of decaying food and mucus. To demonstrate this shrinkage, let a small cube of rubber be vulcanized in a plaster mould, say half an inch in size, carrying the temperature not above 280° or 300°. The time must, of course, be lengthened accordingly. It will be found impossible to do this, and preserve flat sides to the cube. One or more of them will be concaved, unless the temperature has been high enough to render the rubber spongy. By a specific gravity test, the vulcanite will be found to have from three per cent., for pink, to six per cent., for black rubber, more density than the rubber compound before vulcanization.

To overcome the difficulty, if the plate does not call for a very thick layer of vulcanite, use a flask which is closed by spring pressure, and cut a gateway which encircles the plate, with no leaders passing to the cavity of the mould in which the plate is to be formed. Cut away all the surface of the plaster, except a narrow margin, not over an eighth of an inch wide, around the mould. Then the rubber in the mould is imprisoned when the mould is closed, and it becomes very difficult to entirely close the flask. It is not necessary to close it. The spring pressure on the flask will close it as the rubber shrinks in vulcanizing, with the effect of holding the rubber up against the teeth, and preventing the formation of cavities under them.

For very heavy sets, where a greater thickness of vulcanite is necessary, the flask should be propped open at first by inserting thin pieces of sheet metal, from 22- to 30-gauge, and partially vulcanizing the plate. After about half the time for vulcanizing has elapsed, the vulcanizer should be cooled sufficiently to allow of the removal of the flask, the props removed, the flask immediately replaced in the vulcanizer, and the operation finished by continuing the heat, after it has reached the vulcanizing point, for the remainder of the time.

Vulcanizing in steam is preferable to vulcanizing under water, if for no other reason because the plaster does not soften so much.

Geo. B. Snow.

